# IMPACT OF COVID-19 ON HAND AND WRIST ORTHOPEDIC SURGERIES IN A PRIVATE SERVICE

**DOI:** 10.1590/1413-785220253301e276452

**Published:** 2025-02-03

**Authors:** Erick Yoshio Wataya, Katherine Vanessa Tenezaca Rodriguez, Lucas Sousa Macedo, Ricardo Boso Escudero, Luiz Sorrenti, Bernardo Figueira Althoff, Ana Katherina Abarca Herrera, Maurício Pinto Rodrigues, Antonio Carlos da Costa, Mateus Saito, João Carlos Nakamoto

**Affiliations:** 1 Instituto Vita, São Paulo, SP, Brazil

**Keywords:** COVID-19, Hand Injuries, Wrist Injuries, Elective Surgical Procedures, Emergency Treatment, COVID-19, Traumatismos da Mão, Traumatismos do Punho, Procedimentos Cirúrgicos Eletivos, Tratamento de Emergência

## Abstract

**Objective:**

Evaluate the impact of COVID-19 on elective and emergency hand and wrist surgeries operated in a private orthopedic center.

**Methods:**

A retrospective study included hand and wrist surgeries in a private orthopedic center. The total surgeries were computed and separated into elective or emergency surgeries. The numbers were analyzed by month, quarter, and year before and after the pandemic (March 2020).

**Results:**

Eight hundred and forty-three surgeries from March 2018 to February 2022 were included. The mean monthly cases of the initial 12 months of the pandemic (15.3) were statistically equal to previous periods (17.3 and 17.2), but the period from March 2021 to February 2022 showed an increase (20.5; p = 0.037). The first four months of the pandemic had a mean (8.3) lower than the previous period (14.0; p = 0.002), but soon there was a significant increase in the following four months (19.3; p = 0.002). As a historical standard, elective surgeries were greater than an emergency in this institution. Still, in the first two quarters of the pandemic, there was a reduction in elective cases, equaling the emergency.

**Conclusion:**

An important but relatively brief impact on surgical volume was observed in hand and wrist surgeries during COVID-19. A significant reduction in elective cases happened at the pandemic’s beginning followed by a fast recovery after four months. **Nível de Evidência II; Estudo Retrospectivo.**

## Resumo

**Objetivo:**

Avaliar o impacto da COVID-19 em cirurgias eletivas e emergenciais de mão e punho realizadas em um centro ortopédico privado.

**Métodos:**

Foi realizado um estudo retrospectivo que incluiu cirurgias de mão e punho em um centro ortopédico privado. Todas as cirurgias foram computadas e classificadas como eletivas ou de emergência. Os números foram analisados por mês, trimestre e ano antes e depois da pandemia (março de 2020).

**Resultados:**

Foram incluídas 843 cirurgias de março de 2018 a fevereiro de 2022. A média de casos mensais dos 12 meses iniciais da pandemia (15,3) foi estatisticamente igual aos períodos anteriores (17,3 e 17,2), mas o período de março de 2021 a fevereiro de 2022 apresentou um aumento (20,5; p = 0,037). Os primeiros quatro meses da pandemia tiveram uma média (8,3) menor do que o período anterior (14,0; p = 0,002), entretanto, logo houve um aumento significativo nos quatro meses seguintes (19,3; p = 0,002). Como um padrão histórico, as cirurgias eletivas foram maiores do que as emergenciais nessa instituição. Ainda assim, nos dois primeiros trimestres da pandemia, houve uma redução nos casos eletivos, igualando-se à emergência.

**Conclusão:**

Um impacto importante, mas relativamente curto, no volume cirúrgico foi observado nas cirurgias de mão e punho durante a COVID-19. Uma redução significativa nos casos eletivos ocorreu no início da pandemia, seguida de uma rápida recuperação após quatro meses. **Nível de Evidência II; Estudo Retrospectivo.**

## INTRODUCTION

The coronavirus pandemic (COVID-19) has disrupted health services worldwide, testing service’s physical and administrative infrastructure, especially in developing countries.^1^

Orthopedic services, in particular, were obliged to reorganize themselves at all levels of activity, from adopting new safety protocols and use of personal and collective protective equipment to restructuring the flows and processes of all wards of clinics and hospitals, passing through emergency rooms to infirmaries and intensive care units.^2^,^3^,^4^

The demand for orthopedic care during COVID-19 generally decreased during the stricter restriction, but trauma and orthopedic involvement continued to demand attention from services.^5^,^6^ This reduction in cases mainly affected the elective surgeries rate.^3^ Emergency surgeries, such as fractures and infection, continued to occur, but with a reduced number.^7^

The reduction in surgeries also influenced resident physician’s and subspecialist’s training. As a result, school hospitals responsible for education and training had to create alternative ways to meet this lack of demand, such as electronic teaching and telemedicine.^8^,^9^ The objective of this study is to evaluate the impact of COVID-19 on elective and emergency hand and wrist surgeries performed by the Hand Surgery and Microsurgery group in a private orthopedic center in São Paulo – Brazil.

## MATERIAL AND METHODS

A cross-sectional study was performed, with retrospective data collection from the medical records of patients treated at a large private orthopedic service in São Paulo – Brazil, submitted to elective or emergency surgery by the Hand and Microsurgery group from March 2018 to February 2022. This study was evaluated and approved by the Institution Research Ethics Committee (CAAE: 67277423.6.0000.5474).

The number of surgeries performed during COVID-19 (March 2020 to February 2022) was compared with those in the previous two years (March 2018 to February 2020), a period without interference from the pandemic. The data used was age, gender, and date of the procedures performed (elective and emergency) and were analyzed through electronic medical records.

The variables evaluated were presented in tables with absolute and relative frequency distribution. The descriptive analysis was performed in addition to means between the two groups and was compared using the Student’s t-test. A specific three-month moving mean was calculated by the mean number of surgeries from the previous two months and the corresponding month. This mitigates the data by creating a constantly updated mean number and mitigates the impacts of short-term random fluctuations. The significance level adopted was 95%, and the tests were performed in the SPSS software.

## RESULTS

From March 2018 to February 2020 (pre-pandemic period), the total elective surgery was 284, and emergency was 130, totaling 414. Between March 2020 and February 2022 (during the pandemic), the elective surgery was 312, and emergency was 117, totaling 429. Throughout the period (2018–2022), the mean age of the operated patients was 45 years, and 49% were women.

**Figure 1:**
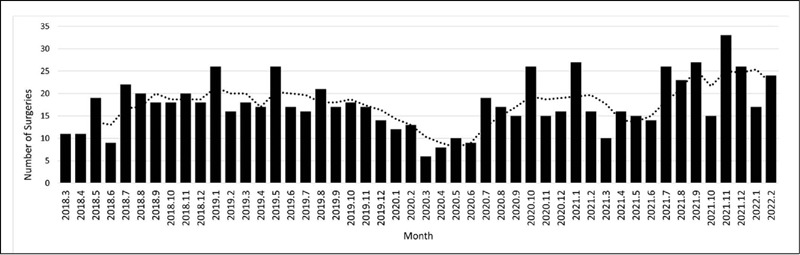
Total number of surgeries performed in different quarters two years before and two years after the onset of COVID-19. The bars represent the number of surgeries, and the dotted line represents the 3-month moving mean.

**Figure 2:**
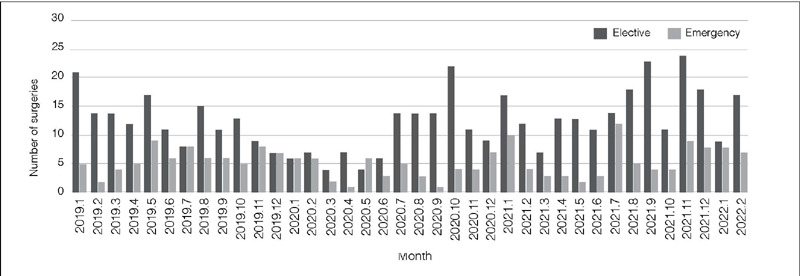
Number of surgeries performed in different quarters two years before and two years after the onset of COVID-19 divided into elective surgeries (dark bars) and emergency surgeries (light bars).

Figure 1 shows the monthly distribution of operated cases, and Figure 2 shows the distribution of surgeries classified as elective or emergency. The qualitative analysis of surgeries throughout the period shows that historically there has been an oscillation in surgeries over the months, but it shows two periods of most considerable reduction, both during the pandemic, between December 2019 and June 2020, and March 2021 and June 2021. The cumulative numbers of surgery performed 12 months before (March 2019 – February 2020) and 12 months after the pandemic (March 2020 – February 2021) show a similarity of elective surgeries (130 vs. 134, respectively) but a reduction in emergency surgeries (76 vs. 50, respectively).

The mean monthly surgeries during the entire period evaluated was 17.6. There was no statistical difference (p = 0.348) between the mean monthly surgeries two years before the pandemic (March 2018 – February 2020), with 11.8 surgeries per month, compared to two years during the pandemic (March 2020 – February 2022), with 13 surgeries per month (Table 1). The month with the lowest number of surgeries was March 2020, with six surgeries. The month with the highest number of surgeries was November 2021, with 27 surgeries. The discriminative analysis between emergency vs. elective surgeries (Figure 2) demonstrated that the elective surgeries was historically higher than emergency, except for November 2018 (9 vs. 11), July 2019 (8 to 8), December 2019 (7 to 7), January 2020 (6 to 6), and May 2020 (4 to 6).

**Table 1:** Comparison between surgeries performed in different periods of four months, one year, and two years, two years before and two years after the onset of COVID-19.

	Mean ± SD	p-value vs. Mar20 – Jun20	p-value vs. Mar20 –Feb21	p-value vs. Previous period
**Period of 4 months**				
Mar18 – Jun18	12.5 ± 4.4	0.062	na	na
Jul18 – Oct18	19.5 ± 1.9	< 0.001	na	0.014
Nov18 – Feb19	20.0 ± 4.3	0.001	na	0.420
Mar19 – Jun19	19.5 ± 4.4	0.001	na	0.438
Jul19 – Oct19	18.0 ± 2.2	< 0.001	na	0.280
Nov19 – Feb20	14.0 ± 2.2	0.002	na	0.003
Mar20 – Jun20	8.3 ± 1.7	-	na	0.020
Jul20 – Oct20	19.3 ± 4.8	0.002	na	0.002
Nov20 – Fen21	18.5 ± 5.7	0.007	na	0.423
Mar21 – Jun21	13.8 ± 2.6	0.006	na	0.090
Jul21 – Oct21	22.8 ± 5.4	0.001	na	0.012
Nov21 – Feb22	25.0 ± 6.6	0.001	na	0.309
**Period of 1 year**				
Mar18 – Feb19	17.3 ± 4.9	na	0.204	na
Mar19 – Feb20	17.2 ± 3.7	na	0.205	0.463
Mar20 – Feb21	15.3 ± 6.6	na	na	0.205
Mar21 – Feb22	20.5 ± 6.9	na	0.037	0.037
**Period of 2 years**				
Mar18 – Feb20	11.8 ± 3.7	na	na	na
Mar20 – Feb22	13.0 ± 5.6	na	na	0.348

SD: Standard deviation

The quarterly analysis of the mean number of surgeries (Table 1) showed that the most critical period in relation to the reduction in surgical volume was the first four months of the pandemic (between March 2020 and June 2020), with a mean of 8.3 surgeries, with a statistical difference (p < 0.05) with all other 4-month periods, except the beginning of 2018 (March 2018 – June 2018, p = 0.062) The analysis of the mean annual surgeries per month (Table 1) showed a tendency to maintain the mean in 2018 (17.3), 2019 (17.2), and 2020 (15.3), with a subsequent increase in 2021, which had a mean monthly of 20.5 surgeries (p = 0.037).

The comparison between the number of elective and emergency surgeries performed in different periods of four months, one year, and two years before and two years after the beginning of COVID-19 is shown in Table 2. All comparisons in the 4-month periods had significant differences, except for November 2019 to February 2020 (p = 0.552), March 2020 to June 2020 (0.144), and November 2021 to February 2022 (p = 0.060). The comparisons year by year and every two years (period before and during COVID-19) were also statistically significant.

**Table 2:** Comparison between the number of elective vs. emergency surgeries performed in different periods of four months, one year, and two years, two years before and two years after the onset of COVID-19.

	Electives Mean ± SD	Urgency Mean ± SD	p-value
**Period of 4 months**			
Mar18 – Jun18	10.3 ± 2.5	2.3 ± 2.6	0.005
Jul18 – Oct18	14.0 ± 2.6	5.5 ± 1.9	0.002
Nov18 – Feb19	14.3 ± 5.0	5.8 ± 3.8	0.037
Mar19 – Jun19	13.5 ± 2.6	6.0 ± 2.2	0.005
Jul19 – Oct19	11.8 ± 3.0	6.3 ± 1.3	0.027
Nov19 – Feb20	7.3 ± 1.3	6.8 ± 1.0	0.552
Mar20 – Jun20	5.3 ± 1.5	3.0 ± 2.2	0.144
Jul20 – Oct20	16.0 ± 4.0	3.3 ± 1.7	0.004
Nov20 – Fen21	12.3 ± 3.4	6.3 ± 2.9	0.037
Mar21 – Jun21	11.0 ± 2.8	2.8 ± 0.5	0.009
Jul21 – Oct21	16.5 ± 5.2	6.3 ± 3.9	0.022
Nov21 – Feb22	17.0 ± 6.2	8.0 ± 0.8	0.060
**Period of 1 year**			
Mar18 – Feb19	12.8 ± 3.7	4.5 ± 3.1	< 0.001
Mar19 – Feb20	10.8 ± 3.5	6.3 ± 1.4	0.001
Mar20 – Feb21	11.2 ± 5.5	4.2 ± 2.6	0.001
Mar21 – Feb22	14.8 ± 5.3	5.7 ± 3.1	< 0.001
**Period of 2 years**			
Mar18 – Feb20	11.8 ± 3.7	5.4 ± 2.5	< 0.001
Mar20 – Feb22	13.0 ± 5.6	4.9 ± 2.9	< 0.001

SD: Standard deviation

## DISCUSSION

COVID-19 interfered with health systems worldwide by prioritizing the treatment of large numbers of patients with often severe clinical respiratory demands and by generating greater attention to prevention measures regarding respiratory isolation in the general population. Consequently, medical services in surgical areas initially significantly reduced admissions and the volume of surgeries in adults and children in several countries.^10^,^11^

This need for a higher attention to the respiratory condition may have also decreased the number of orthopedic surgeries. Blum et al.,^6^ in a systematic review, also concluded that there was a reduction not only in consultations or elective and emergency visits but also in the general trauma surgeries (around 21.2% to 66.7%) and even more in elective surgeries (33.3% to 100%) during the pandemic period. In Brazil, the reduction in surgical volume during the highest period of the pandemic occurred mainly in the Unified Health System (SUS). In nine months there was a reduction of about 46% in elective surgeries attributed to COVID-19.^12^

As demonstrated in our study, during the first two years of COVID-19 in Brazil, considering the reality of the service in question, there was an important change in the flow of surgeries in the hand surgery subspecialty, but only occasionally, in the first four months following the decree of the World Health Organization that confirmed that we were facing a pandemic.

**Figure 3:**
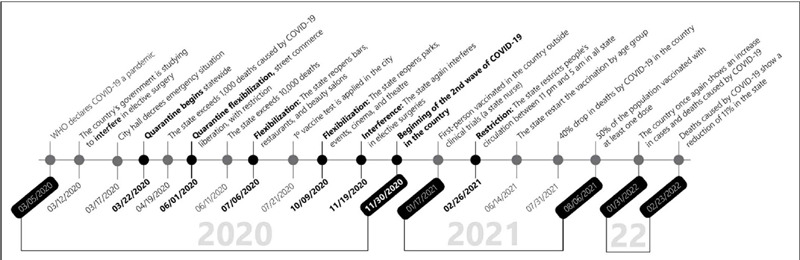
COVID-19 timeline in the state where work was performed.

Certainly, the reasons for certain services to present a more or less significant reduction in the surgical volume were diverse and heterogeneous. However, we can draw a parallel between the surgical volume curve and the epidemiological situation experienced in the city where the work was performed (Figure 3). This analysis suggests an inversely proportional relationship between the periods of lower volume of surgeries with the period of stricter restriction.

The reduction in the mean monthly surgeries in the first four months of the pandemic was similar to other studies.^5^,^6^ However, in our study, this tendency did not continue in the subsequent months, including a demand similar to the two pre-pandemic years. Possibly, the fact that our institution did not directly serve patients with respiratory demand, combined with the effectiveness of organizational measures and administrative flows, made it possible that the reduction in surgical volume did not occur sustainably. In addition, the type of health service may have had a great influence on the rapid recovery of these numbers since it may have been seen as a place of safety for patients with orthopedic demand who did not want to be exposed to hospitals that were treating cases of COVID-19.^2^

The higher period of surgeries in the institution among the four years evaluated was after July 2021. We can also contextualize with the local health scenario, which at that time was experiencing a gradual reopening and was heading for a vaccination rate (1st dose) of 50% of the eligible population, which occur in August 2021, reaching the mark of 80% of the country’s population at the end of December 2021. The timeline shown in Figure 3 illustrates the epidemiological scenario of COVID-19 in the state where this study was performed, the first and most affected by the pandemic in the country.

The limitations of this study start with the specificity of the study in a single center, so we must be careful in extrapolating these results. Still, we must remember that the occasional fluctuation in the number of cases can also occur due to other factors, population vacation periods, and habitual seasonality of certain pathologies, among others.

## CONCLUSION

Our study showed a significant reduction in surgeries at the beginning of the pandemic, with elective surgeries most affected. However, after four months, there was already a quick recovery, and the numbers were re-established. COVID-19 was a major organizational challenge for health services in all countries. However, we showed a rapid recovery in a private institution with exclusive performance in orthopedics, showing the importance of the flow of the demands of the cases studied.
